# Preclinical antibody-PET imaging of PD-L1

**DOI:** 10.3389/fnume.2022.953202

**Published:** 2022-08-09

**Authors:** Emma L. Brown, Rachel A. DeWeerd, Abbey Zidel, Patricia M. R. Pereira

**Affiliations:** ^1^Department of Radiology, Mallinckrodt Institute of Radiology, Washington University School of Medicine, St. Louis, MO, United States; ^2^Division of Biology and Biomedical Sciences, Washington University School of Medicine, St. Louis, MO, United States

**Keywords:** antibody-PET, PD-L1, immune checkpoint inhibition, preclinical PET imaging, heterogeneity

## Abstract

Programmed cell death protein-1/ligand-1 (PD-1/PD-L1) blockade, including antibody therapeutics, has transformed cancer treatment. However, a major challenge in the field relates to selecting patients who are likely to respond to immune checkpoint inhibitors. Indeed, biopsy-based diagnostic tests to determine immune checkpoint protein levels do not accurately capture the inherent spatial and temporal heterogeneity of PD-L1 tumor expression. As a result, not all PD-L1-positive tumors respond to immunotherapies, and some patients with PD-L1-negative tumors have shown clinical benefits. In 2018, a first-in-human study of the clinically-approved anti-PD-L1 antibody Atezolizumab labeled with the positron emitter zirconium-89 validated the ability of positron emission tomography (PET) to visualize PD-L1 expression *in vivo* and predict tumor response to immunotherapy. These studies have triggered the expansion of PD-L1-targeted immunoPET to assess PD-L1 protein levels and PD-L1 expression heterogeneity in real time and across the whole tumor. First, this mini-review introduces new PD-L1 PET imaging studies of the last 4 years, focusing on the expansion of preclinical tumor models and anti-PD-L1 antibodies/antibody fragments in development. Then, the review discusses how these preclinical models and targeting agents can be utilized to study spatial and temporal heterogeneity of PD-L1 expression.

## Introduction

Immune checkpoint inhibition is a mainstay in cancer treatment (1). The regulatory checkpoint pathway involving the programmed cell death protein 1 (PD-1) and its ligand programmed cell death ligand 1 (PD-L1) is a major mechanism for cancers to escape immune attack. This pathway includes PD-1 on T cells and PD-L1 on cancer cells and antigen-presenting cells. Cancer cells upregulate cell-surface PD-L1 levels to increase PD-1-mediated inhibitory signaling, and antibodies that target PD-L1 have shown impressive clinical outcomes for multiple cancers (1). At the time of this review, there are three clinically-approved antibodies specific for PD-L1: Atezolizumab, Avelumab, and Durvalumab (2, 3). In addition to being used as therapeutics, anti-PD-L1 antibodies labeled with positron emitters can be employed as companion diagnostics and allow assessment of PD-L1 protein levels and therapeutic response in patients with cancer (4). For this mini-review, we focus on preclinical PD-L1-targeted immuno-positron emission tomography (PET) imaging reported in the last 4 years.

The expansion in PD-L1 immunoPET has been triggered by the ability of this non-invasive technology to provide information complementary to conventional methods of immunohistochemistry (IHC) used to identify patients that will likely benefit from PD-L1 immunotherapies. Indeed, the extensive heterogeneity of PD-L1 expression across the entire tumor volume results in inaccurate patient stratification when using minimal tissue biopsies for IHC (5–7). Immuno-PET has demonstrated great promise in determining protein levels and heterogeneity of immune checkpoint PD-L1 (2), which allows patient stratification, prediction and monitoring response to immune checkpoint inhibitors non-invasively across a patient's therapy regime.

In 2018, two landmark publications showed the feasibility and safety of radiolabelled agents targeted toward PD-L1 (8, 9) to non-invasively image its expression in human tumors and healthy tissues. For information on considerations for the development and use of PD-L1 tracers in the clinic and early preliminary work conducted prior to 2018, we refer the reader to other reviews (2, 3). Here, we concisely review preclinical PD-L1 PET imaging research, which has followed the landmark publications of 2018. We first discuss the expansion of preclinical PD-L1 imaging antibody and antibody-fragment based imaging agents, where an increasing number of models and targeting agents are being developed. We then describe studies illustrating the potential of preclinical molecular PD-L1 imaging to reveal PD-L1 biology, specifically discussing the use of preclinical imaging to monitor spatial and temporal heterogeneity of PD-L1 expression throughout tumor development and treatment regimes.

## Expansion of preclinical PD-L1 molecular imaging

Since the first-in-human trials utilizing radiolabeled antibodies targeted toward PD-L1/PD-1 were reported (8, 9), clinical imaging [recently reviewed by Hegi-Johnson et al. (3)] of PD-L1 expression in human cancer has expanded. In the preclinical setting, the number of targeting agents and models used is increasing, demonstrating excellent specificity and imaging capabilities, and illustrating the potential of non-invasive *in vivo* PD-L1 molecular imaging across multiple cancer types. These preclinical studies are concisely outlined below and reported in Table 1.

**Table 1:** Preclinical imaging studies of antibody/fragment PET imaging agents for PD-L1 that have been reported in the last 4 years.

	**Antibody/label**	**Mice strain/cell line**	**Tumor accumulation (%ID/g)**	**Biodistribution**	**Reference**
**Full-Length Antibodies**	^89^Zr-DFO-C4 ^89^Zr-DFO-Atezolizumab	Athymic nu/nu, C57BL/6J. B16 F10 PD-L1^high^ H1975 PD-L1^moderate^	^**89**^**Zr-C4** B16 F10: 13.83 ± 0.50 H1975: 7.08 ± 0.8 ^**89**^**Zr-Atezolizumab** B16 F10: 13.92 ± 1.0 H1975: 3.97 ± 1.0	^**89**^**Zr-C4 in C57BL/6J** Liver: 7.33 ± 1.1 Spleen: 6.05 ± 0.2 Kidney: 2.76 ± 0.8 ^**89**^**Zr-atezolizumab in C57BL/6J** Liver: 6.79 ± 1.6 Spleen: 19.95 ± 1.5 Kidney: 6.71 ± 0.3	(11)
	^89^Cu-NOTA-MX001	C57BL/6, BALB/c, Nu/Nu. MC38 PD-L1^high^ BGC823 PD-L1^high^ 4T1 PD-L1^low^ U87MG PD-L1^low^ [expression confirmed by FACs and IHC]	**C57BL/6 mice bearing MC38 tumors, 48 h** 15.06 ± 4.52	**C57BL/6 mice bearing MC38 tumors, 48 h** Liver: 12.35 ± 1.27 Spleen: 6.37 ± 0.48 Kidney: 4.39 ± 1.13	(24)
	^89^Zr-DFO-Atezolizumab	NOD/SCID, and patient derived xenograft of clear cell renal carcinoma (ccRCC). [PD-L1 expressed in >30% tumor cells by IHC]	**Day 6 post-injection** Independent cohort 1: 4.2 ± 0.6 Independent cohort 2: 5.2 ± 0.4	Tumor/muscle ratio: 4.4 ± 0.4	(10)
	^89^Zr-DFO-Avelumab	Nu/nu MDA-MB-231 xenografts [PD-L1 moderate by *in vitro* binding study]	**Day 1 (D1) post-injection:** 2.79 ± 0.3 **D3**: 2.93 ± 0.54 **D7**: 2.44 ± 0.58	**Liver:** D1-20.08 ± 3.25 D7-10.93 ± 3.04 **Spleen:** D1- 60.41 ± 6.23 D7- 29.90% ± 7.58 **Kidney:** D1-10.26 ± 1 D7- 5.06 ± 0.81 **Lymph nodes:** D1-18.64 ± 1.6 D7- 27.03 ± 5.38	(13)
	^89^Cu-NOTA-Anti-PD-L1	C57BL/6; murine pancreatic cells with KRAS^G12D^ mutation injected orthopically.	**24 h post-injection** >10%ID/g	**Highest to lowest %ID/g at 24 h post-injection:** Lymph node, spleen, liver, kidney	(22)
	^89^Zr-CX-072	C57BL/6, BALB/c. MC38 PD-L1^low^, MDA-MB-231 PD-L1^high^. [expression confirmed by flow cytometry]	**BALB/c mice bearing MDA-MB-231 tumors, accumulation in spleen at 6 days post-injection** 10 μg labeled: 8.7 ± 1; 10 μg labeled + 40 μg unlabeled: 6.0 ± 1.3; 10 μg labeled + 240 μg unlabeled: 4.3 ± 0.7	**BALB/c mice bearing MDA-MB-231 tumors, accumulation in spleen at 6 days post-injection:** 10 μg labeled: 25.8 ± 4.1 10 μg labeled + 40 μg unlabeled: 10.8 ± 2.8 10 μg labeled + 240 μg unlabeled: 5.3 ± 2.6	(19)
	^89^Zr-DFO-Avelumab	Nu/Nu MDA-MB-231 xenografts [PD-L1 expression validated by immunofluorescence and FACs]	**48 h post-injection** **Targeting group:** 6.1 ± 1.0 **Pre-blocked group:** 5.2 ± 1.0	**At 48 h (unblocked)** Spleen: 10.2 ± 0.7 Lymph nodes: 6.9 ± 1.0 **At 48 h (blocking study)** Spleen: 4.9 ± 0.5 Lymph nodes: 5.8 ± 1.1	(12)
	^89^Zr-DFO-6E11	NMRI nude, BALB/c, C56BL/6. NSCLC lines (H1703- PD-L1^low^, H1993- PD-L1^moderate^, HCC827- PD-L1^high^), CT26.WT- PD-L1^high^, and B16F10- PD-L1^high^ [FACs, confirmed by IHC]	**Mean tumor uptake, 72 h** H1703: 1.35 ± 0.1; H1993: 2.32 ± 0.2; HCC827: 5.1 ± 0.6; CT26: 8.26 ± 0.6; B16F10: 10.78 ± 0.9.	**HCC827 in NMRI nude mice, 144 h (unblocked)** Liver: 7.99 ± 0.03 Spleen: 14.44 ± 3.1 Kidney: 2.11 ± 0.28 Tumor*:* 0.35 ± 0.04 Tumor/blood ratio: 9.01 ± 1.82 Tumor/muscle ratio: 1.25 ± 0.05	(21)
	^89^Zr-DFO-anti-6E11	C57/BL6J KPC cells injected orthotopically into pancreas	**At 72 h, vehicle:** <5%ID/g **At 72 h, ERKi:** ~10%ID/g	**Vehicle cohort (highest to lowest %ID/g):** spleen, liver, lungs, blood/kidney, bone **ERK inhibition cohort (highest to lowest %ID/g):** spleen, liver, lungs, blood/kidney, bone	(20)
	^89^Zr-DFO-REGN3504	SCID PD-1/PD-L1 humanized mice NCI-H441, MDA-MB-231, HCC827 [moderate PD-L1 expression by FACs and low by IHC)] MC-38 with CRISPR KO of mPD-L1 and engineered to express hPD-L1. All lines implanted into flank of mouse Primate model: cynomolgus monkey	**NCI-H441:** 52.3%ID/g **HCC827:** 38.7%ID/g **MDA-MB231:** 38.8%ID/g	**Humanized mice, MC-38/hPD-L1 tumors, Vehicle cohort** Spleen: 29.3 ± 1.8 Inguinal lymph nodes: 4.5 ± 8.0 Brachial lymph nodes: 28.8% ± 7.4 **Clodronate treated** Spleen: 8.9 ± 4.5 Inguinal lymph nodes: 24.5 ± 8.0 Brachial lymph nodes: 18.7 ± 3.9	(23)
	^124^I -Durvalumab	Male SPF Balb/c nude A549 (PD-L1^low^) and H460 (PD-L1^high^) [PD-L1 expression validated by qPCR, western blot, flow cytometry, IHC]	**48 h post-injection:** 5.18 ± 0.73% ID/g	**12 h post-injection:** Blood: 22.01 ± 1.34%ID/g Kidneys: 4.68 ± 0.48% ID/g **48 h post-injection:** Blood: 17.84 ± 0.82% ID/g **72 h post-injection:** Blood: 16.34 ± 1.08% ID/g **Tumor-to-liver ratio (after 12 h post-injection):** >2	(17)
**Antibody Fragments**	^89^Zr-DFO-KN035	BALB/c nude LN229 (PD-L1^high^ by IHC), A375	**24 h post-injection:** 18.08 ± 2.34 **120 h post-injection:** 4.66 ± 0.93	**In LN229 tumor bearing mice** **24 h post-injection**	(29)
		(engineered to express PD-L1)		Kidneys: 8.16 ± 1.84 Liver: 7.36 ± 0.32 Spleen: 2.83 ± 0.41 Blood: 13.21 ± 1.86 Tumor:blood- 0.5 ± 0.05 Tumor:muscle- 5.64 ± 0.65 **120 h post-injection** Kidney: 6.69 ± 0.94 Liver: 5.69 ± 0.58 Spleen: 2.84 ± 0.76 Blood: 4.2 ± 0.36 Tumor/blood: 1.1 ± 0.12 Tumor/muscle: 7.7 ± 1.37	
	^89^Cu-NOTA-anti-PD-L1 (Fab conjugate)	Athymic nude C57BL/6	**n/a**	**Nude mice**, ***in vivo*** **45 min post-injection** BAT: 6.1 ± 2.1 Spleen: 12.0 ± 1.9 **C57BL/6 mice**, ***ex vivo*** **90 min post-injection** Kidneys: 157 ± 27 BAT: 4.5 ± 1.5 Spleen: 9.4 ± 3.9	(26)
	^68^Ga-NOTA-Nb109	BALB/c nude A375 (PD-L1^low^), A375-hPD-L1 (transfected, hPD-L1^high^), MCF-7 (PD-L1^low^)	**1 h post-injection** A375-hPD-L1: 5.0 ± 0.35 MCF-7: 1.7 ± 0.36 **2 h post-injection** A375-hPD-L1: 4.05 ± 0.31 MCF-7: 1.46 ± 0.34	**A375-hPD-L1 tumor bearing mice, 1 h post-injection** Kidneys: 33.66 ± 3.26 Liver: 1.11 ± 0.41 Remaining organs: <1.5%ID/g Tumor/blood: 5.48 ± 0.12 Tumor/muscle: 9.33 ± 0.82 **2 h post-injection** Tumor/blood: 7.07 ± 0.11 Tumor/muscle: 6.76 ± 0.41	(27)
	^89^Zr-Df-F(ab')_2_	C57BL/6 B16F10 PD-L1^high^ by flow cytometry	**From graph, 2 h post-injection:** ~4%ID/g	**2 h post-injection:** Kidney: ~12%ID/g Liver: ~8%ID/g	(28)
	^124^I -Durvalumab- F(ab')_2_	Male SPF Balb/c nude A549 (PD-L1^low^) and H460 (PD-L1^high^) [PD-L1 expression validated by qPCR, western blot, flow cytometry, IHC]	**12 h post-injection:** 5.29 ± 0.42% ID/g	**12 h post-injection:** Blood: 15.41 ± 1.49%ID/g Kidneys: 7.60 ± 0.59% ID/g **48 h post-injection:** Blood: 6.01 ± 0.62% ID/g **72 h post-injection:** Blood: 5.07 ± 0.29% ID/g **Tumor-to-liver ratio (after 4 h post-injection):** >2	(17)

89 Zr, Zirconium-89;

64 Cu, Copper-64;

68 Ga, Gallium-68;

PD-L1, Programmed death-ligand 1; NOTA, 1,4,7-triazacyclononane-1,4,7-triacetic acid; DFO, Deferoxamine; ID, injected dose; BAT, brown adipose tissue.

### Clinically-approved anti-PD-L1 antibodies

The combination of antibodies' specificity with the sensitivity of PET allows measurements of *in vivo* PD-L1 expression in both the laboratory and clinical settings. Since the publication of the first-in-human zirconium-89 (^89^Zr)-labeled Atezolizumab study in 2018, radiolabeled Atezolizumab and Avelumab, which bind both human and mouse PD-L1, have been explored preclinically in different tumor models (Table 1).

Atezolizumab, an anti-PD-L1 antibody with a mutant Fc, showed accumulation in two independent clear cell renal carcinoma xenografts derived from the same patient (10) (4.2 ± 0.6%ID/g and 5.2 ± 0.4%ID/g, Table 1), which mimicked uptake values in a human lung cancer model (11) (3.97 ± 1%ID/g, Table 1). In a syngeneic mouse model, melanoma tumors with high PD-L1 expression showed correspondingly higher Atezolizumab uptake in the tumor, compared to the aforementioned models (13.92 ± 1.0%ID/g, Table 1).

Avelumab has been investigated in multiple independent breast cancer xenograft cohorts, showing tracer uptake of 6.1 ± 1%ID/g (12) and 2.93 ± 0.54%ID/g (13) (Table 1). In a dose escalation study by Jagoda et al. (13) co-injection of unlabeled Avelumab led to higher tumor uptake values with decreased uptake in other organs such as spleen. Blocking with unlabeled or “cold” antibodies is a method often used to limit off-target interactions with healthy organs that express PD-L1 and Fc-gamma receptors. The importance of optimizing dose and time-points of admnistration, the specific activity of radiolabeled antibodies, and the amount of labeled and unlabeled antibody to be injected to reduce off-target uptake while preserving tumor targeting are reviewed elsewhere (14, 15).

Studies using radiolabeled Durvalumab as an imaging agent have only recently begun, with the first clinical study published in 2022 (16) and others underway (14). Preclinical studies using Durvalumab as a PD-L1 imaging agent are limited (17) probably due to the fact that Durvalumab interacts exclusively with human PD-L1 and mouse models may be unable to recapitulate human biodistribution of the antibody.

### Novel full-length anti-PD-L1 antibodies

In addition to the preclinical expansion of currently approved antibodies, novel full-length antibodies are being developed. In a comparative study of Atezolizumab and a newly developed C4 anti-PD-L1 antibody, Moroz et al. found that C4 distinguishes PD-L1 positive tumors while maintaining lower background in healthy organs than Atezolizumab in immunocompetent mice (tumor: 13.83 ± 0.5 C4 *vs*. 13.92 ± 1 Atezolizumab; liver: 7.33 ± 1.1 C4 *vs*. 6.79 ± 1.6 Atezolizumab; spleen: 6.05 ± 0.2 C4 *vs*. 19.95 ± 1.5 Atezolizumab; kidney: 2.76 ± 0.8 C4 *vs*. 6.71 ± 0.3%ID/g Atezolizumab, Table 1) (11, 18).

In other works, a protease-activatable antibody, CX-072, showed specific tumor targeting once activated by proteases in the tumor microenvironment (TME) (19). Here, uptake in breast tumor xenografts and spleen was 2.1-fold higher and 5.3-fold lower, respectively, when using the activatable antibody *vs*. control (non-activatable) antibody. The validation of CX-072 as an imaging agent for patients with solid tumors or lymphomas was further explored in the PROCLAIM-CX-072 clinical trial (NCT03013491) (19).

Other antibodies in development include the antibody clone 6E11 (Genentech), which has been investigated in mouse models of non-small cell lung cancer and pancreatic ductal adenocarcinoma (20, 21). In the pancreatic tumor model, high uptake was noted in the spleen and liver (~3–4 fold higher *vs*. tumor), as well as blood and lungs (20) (Table 1). For abdominal tumors such as pancreatic tumors, co-injecting unlabeled antibody at a dose that reduces radiolabeled antibody accumulation in the spleen may facilitate tumor visualization (22). In the non-small cell lung cancer model, Christensen et al. (21) observed similar uptake of 6E11 in spleen, liver, and lungs, with notably lower 6E11 levels in blood. Co-injection of unlabeled 6E11 showed a decrease in 6E11 uptake in spleen and liver with an overall tumor uptake of 3.07 ± 0.15% ID/g.

Another antibody in development, REGN3504 (Regeneron), has been examined in breast and lung cancers, showing high tumor uptake (up to 50%ID/g, depending on tumor type, Table 1) as well as binding to spleen and lymph nodes (29.3 ± 1.8%ID/g in spleen, 24.5 ± 8.0%ID/g in inguinal lymph nodes, 28.8 ± 7.4%ID/g in brachial lymph nodes, Table 1) (23). These novel antibodies demonstrate potential to monitor PD-L1 expression with high specificity across multiple tumor types (11, 19–24).

### Anti-PD-L1 antibody fragments

In addition to full-length antibodies, smaller antibody fragments (~15–80 kDa) allow for accelerated targeting and clearance while retaining high PD-L1 specificity (25). To this point, several of the antibody fragments presented in Table 1 show tumor uptake within 2 h after antibody administration (26–28). High uptake is also observed in the kidneys (26–28), owing to the faster renal clearance of these small molecules compared to full-length antibodies. Furthermore, PET images of the single-domain antibody KN035 at 24 h post-injection demonstrated high tumor uptake in a human glioma murine model and lower accumulation in the kidneys compared to fragments imaged closer to the time of injection (Table 1) (29). KN035 is currently being investigated in numerous clinical trials from phase 1 to 3 and may be a promising fragment for clinical imaging setting (NCT04977128 and NCT03638804). Beyond targeting the primary tumor region, a very recent study demonstrated the potential of iodine-124 labeled anti-PD-L1 fragments to offer good tumor-to-background contrast for visualizing metastases, with minimal radionuclide shedding to bone and tumor-to-liver ratios of 2 in non-small cell lung cancer (17).

### Preclinical models of PD-L1 expressing tumors

In order to investigate the ability of PD-L1 antibodies and fragments to interact with human PD-L1 on human tumor cells, preclinical studies include PD-L1 overexpressing cell lines (27, 29). It should be noted that these overexpression models may not reflect clinical PD-L1 heterogeneity and protein levels and may overestimate the uptake of the anti-PD-L1 imaging agents in human tumors. To address this, Table 1 describes studies that have used multiple models in addition to those which overexpress PD-L1 to affirm the possibility of translatable imaging (29).

Preclinical research has also relied heavily on subcutaneous injection of human cancer cell lines into immunocompromised mice. While immunocompromised mice demonstrate tumor-binding ability and specificity, it is important to recognize that PD-L1 is also expressed on host cells, as well as in other murine tissues (30, 31). Additionally, PD-1, the co-inhibitory receptor to PD-L1, is highly expressed on T-cells which are absent in many of these immunocompromised models. Thus, the binding of PD-L1 antibodies/fragments to local and circulating immune cells, or other cells in the body, will need to be investigated further to ensure tumor targeting without excessive host background. In fact, PD-L1 expression is high in tumor-associated macrophages, and uptake of PD-L1 targeting agents by macrophages will also influence biodistribution and should be thoroughly characterized (32). To study the possible uptake of anti-PD-L1 antibody by murine macrophages, Kelly et al. (23) performed biodistribution studies in mice upon chemical depletion of macrophages. Clodronate-induced macrophage depletion decreased splenic and lymph node uptake, although uptake was still relatively high in these organs (8.9 ± 4.5%ID/g in spleen and ~18.7–19%ID/g in lymph nodes, Table 1) without impacting tumor uptake. The same group demonstrated that anti-PD-L1 antibody accumulates in the spleen, lymph nodes, thymus, and liver of mice genetically humanized to express human PD-L1 and PD-1 (23). Interestingly, localization to spleen and lymph nodes occurs across immunocompromised and immunocompetent models used in the studies discussed here (Table 1), suggesting that immunocompromised models may still serve as useful tools when initially investigating PD-L1 imaging agents, perhaps due to a degree of functioning innate immunity. Nevertheless, the use of transgenic or syngeneic models alongside immunocompromised models where possible may provide more realistic and clinically applicable settings for antibody/fragment analysis.

## Revealing PD-L1 spatial and temporal heterogeneity with molecular imaging

The studies described thus far in this review demonstrate the ability to target PD-L1 with molecular imaging agents. Having shown that this is possible across multiple cancer types, animal models and with multiple radiolabeled targeting agents now available, this section draws on how non-invasive information of PD-L1 status can be utilized to reveal aspects of PD-L1 biology, particularly its inherent spatial and temporal heterogeneity.

### Antibody-PET of PD-L1 heterogeneity

Heterogeneity of PD-L1 expression is widely observed in tumor specimens and is of clinical concern when considering PD-L1 protein levels as a biomarker to stratify patients for checkpoint inhibitor therapy (5–7). This heterogeneity is 2-fold: spatial and temporal. Spatial heterogeneity of PD-L1 expression causes sampling bias in the clinic, as a small patient biopsy will not necessarily represent expression across the entire tumor tissue (33). Temporal heterogeneity of PD-L1 expression across disease progression and therapy regimes is also reported, with some tumors initially negative and then becoming positive for PD-L1 (34), which represents another clinical challenge as acquiring numerous patient biopsies is not always possible. Molecular imaging of PD-L1 across entire tumor volumes may provide a complementary tool to a clinical oncologist to non-invasively monitor both spatial and temporal heterogeneity. Importantly, the molecular mechanisms which give rise to this heterogeneity are not well described and are largely unknown. Additionally, while mechanisms of spatial and temporal heterogeneity are uncovered, visualizing and monitoring them accurately is limited with the current clinical standard of IHC and assays conducted on minimal tissue biopsies.

### PD-L1 spatial heterogeneity

In the landmark Bensch et al. study, PD-L1 on IHC co-registered with autoradiography of radiolabeled antibody uptake, as these patients had previously undergone ^89^Zr-Atezolizumab PET imaging (8). Similar studies were conducted preclinically in human breast tumor xenografts with the novel protease-activatable ^89^Zr-CX-072 (19). In both studies, areas of high and low PD-L1 expression on IHC spatially correlated with uptake of radiolabeled antibody on autoradiography, helping to validate the molecular imaging agent.

**Figure 1:**
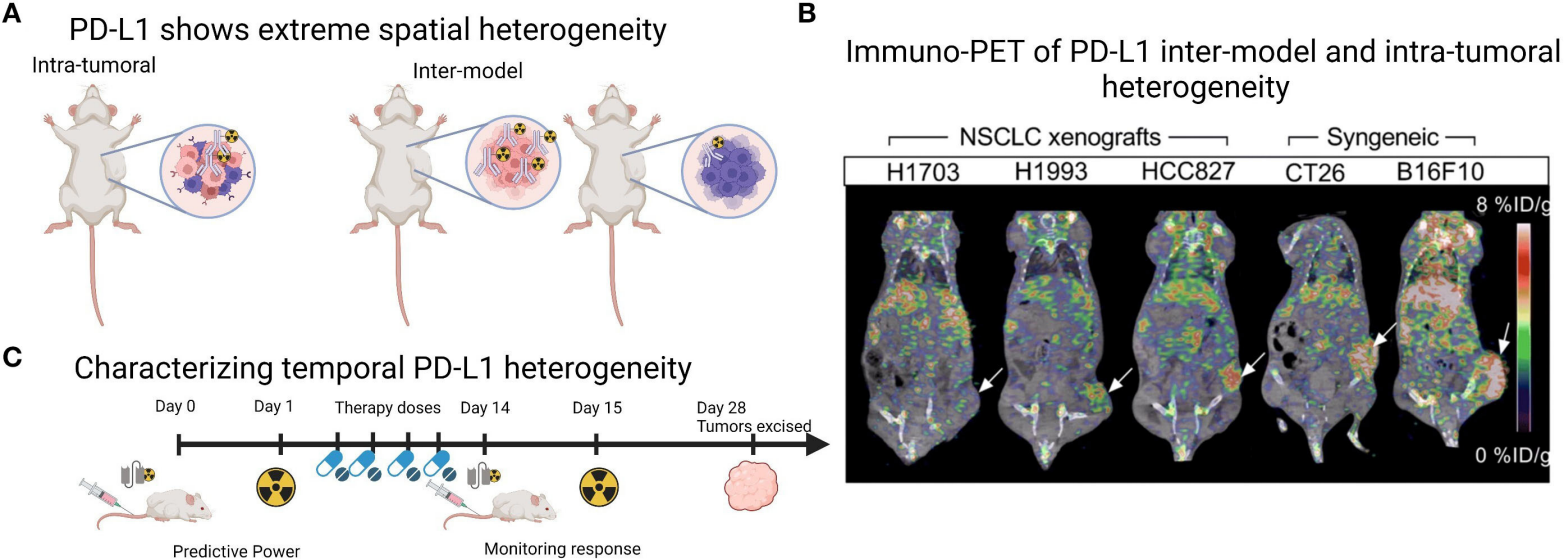
Imaging PD-L1 heterogeneity with PET imaging. **(A)** Schematic demonstrating that preclinical tumor models show both intra-tumoral and inter-model heterogeneity which can be monitored with radiolabeled PD-L1 targeting agents. **(B)** Example of PET images from Christensen et al. (21) demonstrating inter-model and intra-tumoral heterogeneity across human xenografts ranging from low to high PD-L1 expression in the tumor region (white arrow) and across two syngeneic mouse-derived cell line xenografts CT26 (colon carcinoma, white arrow) and B16F10 (melanoma, white arrow). Image under a Creative Commons Attribution 4.0 International License, no changes were made to the image. To view a copy of this licence, visit http://creativecommons.org/licenses/by/4.0/.CC BY license. **(C)** Schematic demonstrating that PD-L1 PET imaging agents, particularly antibody fragments, could be utilized multiple times across a treatment regime, as PD-L1 PET may provide predictive power or monitor response to treatment. Panles (**A** and **C**) created with BioRender.com.

A current challenge for mouse models of human cancer is accurate recapitulation of the TME, which may impede antibody delivery to the tumor. Cells within the TME, particularly other immune cells and stromal cells, as well as physical barriers such as fibrosis and vascular perfusion, may impact the ability of PD-L1 targeting agents to bind their antigen. Of the studies presented in Table 1, two utilized an immune-competent orthotopic pancreatic ductal adenocarcinoma (PDAC) *LSL-Kras*^*G*12*D*/+^*;LSL-Trp53*^*R*172*H*/+^*;Pdx-1-Cre* (KPC) model, to more accurately recapitulate the pancreatic TME (20, 22). In this model, the addition of an Erk inhibitor to increase PD-L1 levels significantly increased tumor uptake of the full-length antibody 6E11 (compared to vehicle treated control), suggesting Erk inhibition can prime the TME for PD-L1 targeted therapies (20). Additionally, a hypoxic TME is known to increase the expression of PD-L1 (35). Indeed, oncogenic pathways such as epidermal growth factor receptor signaling regulate PD-L1 expression (33), while chemo-, radio- and antibody-therapeutic regimes are reported to increase PD-L1 expression (34, 36). While many of the studies in Table 1 displayed inter-model heterogeneity in PD-L1 expression, they also showed intra-tumoral spatial heterogeneity across the entire tumor volumes in PET imaging (Figures 1A,B) (12, 21, 27, 29), and recent progress in computational approaches will be helpful to understand this complex heterogeneity of PD-L1 (37). While investigating PD-L1 heterogeneity preclinically, PD-L1 PET images could be spatially colocalized to large tissue areas processed *ex vivo* in IHC or similar methods, to co-register areas of high uptake with molecular expression.

While not discussed in this mini-review, PD1, the co-inhibitory receptor of PD-L1, expressed on T-cells, is also a target of checkpoint inhibition and shows extreme spatial heterogeneity on IHC and in molecular imaging (9, 38). As the two are so heavily related, monitoring the expression of both PD1 and its ligand PD-L1 may prove useful in a clinical setting. We therefore direct the reader to the other reviews for further information on PD1 imaging (2, 14).

### PD-L1 temporal heterogeneity

Temporal heterogeneity of PD-L1 expression could be explored with PD-L1 molecular imaging, particularly in preclinical studies that mimic clinical treatment regimes, with a view to translate these findings to clinical applications. So far, preclinical studies have largely focused on monitoring response to standard therapies, showing an increase in PD-L1 uptake and immune activation in response to radiotherapy and immunotherapy (21, 39, 40), corroborated by *ex vivo* IHC. The predictive value of PD-L1 PET imaging is also demonstrated by Christensen et al. (21) in a study showing that PD-L1 PET signal (expressed as maximum tumor to muscle ratios) following radiotherapy treatments negatively correlated with tumor volume increase. Where along the treatment pathway PD-L1 PET would be most predictive or provide accurate measurement of response is currently unclear and could be explored in preclinical treatment regimes. Additionally, utilizing the non-invasive nature of molecular imaging to include multiple imaging time-points longitudinally would help to characterize temporal heterogeneity. Here, the potential role of antibody fragments labeled with short half-life radionuclides comes to the forefront, as their fast clearance and decay would enable multiple imaging time-points for the same tumor within a treatment period of a few months (Figure 1C) (25). Additionally, there are multiple small peptide fragments in development for PD-L1 imaging (14), a discussion of which was largely beyond the scope of this review. In this context, the fragment DK222 was able to determine PD-L1 availability at the cell surface, which could then be targeted with anti-PD-L1 antibody therapy. Target availability was shown to relate to tumor volume increase across the treatment period in breast and melanoma models (40). Both the predictive power and ability to monitor response to therapy of PD-L1 PET imaging will likely continue to be explored in the coming years (Figure 1C).

## Discussion

Checkpoint inhibitor therapy has been heralded as a game-changer to treat multiple solid tumors (1). While durable responses can be seen in many patients, response rate can be as low as 15% (1). Therefore, monitoring the expression of checkpoint inhibitor ligands such as PD-L1 is essential to stratify patients that will respond to these therapies as well as monitor response to treatment. Currently, PD-L1 protein levels are monitored by IHC in minimal *ex vivo* tissue biopsies. A disadvantage of measuring PD-L1 expression in tumor samples *via* IHC is that this can only be conducted on a small section of the tumor tissue taken during a single biopsy, disregarding the extensive spatial and temporal heterogeneity of PD-L1 that exists (33). Additionally, PD-L1 IHC diagnostic tests are often discordant, and range in their threshold of PD-L1 positivity (>1% or >50%) (41, 42). Indeed, many of the publications quoted in Table 1 are discordant in their classification of PD-L1 high and low tumors by IHC or *ex vivo* methods.

Molecular imaging of PD-L1 using radiolabeled antibodies or antibody fragments presents a non-invasive approach to visualize expression of PD-L1 across the entire tumor volume. Since the first-in-human studies of molecular imaging of PD-L1 were published in 2018 (8, 9), there has been an expansion in the number of targeting agents proposed and tested across multiple cancer types (Table 1). In this review, we outlined how novel agents and models have been tested preclinically, focusing on both antibodies and antibody fragments that are under development (Table 1). These studies demonstrated a range of tumor uptake values, as well as uptake in other lymphatic organs including the spleen and lymph nodes. Having demonstrated the ability to target PD-L1 expression in these models, these studies can inform human dosimetry as these imaging agents move toward the clinic. However, caution should be taken with the heavy-reliance thus far on immunocompromised models and PD-L1 overexpressing cell lines, and the use of syngeneic and transgenic models alongside these will provide more accurate modeling of biodistribution and the TME. We believe the current landscape of preclinical PD-L1 molecular imaging presents a useful platform for translational studies, with many targeting agents and animal models available for research.

We discussed how these preclinical models could be utilized to further explore PD-L1 spatial and temporal heterogeneity. Owing to the fact that molecular imaging captures the entire tumor volume and is non-invasive, we present PD-L1 molecular imaging as a possible complementary tool for a clinical oncologist to determine patient PD-L1 expression. From a preclinical perspective, molecular imaging would be utilized in determining the origins of spatial and temporal heterogeneity of PD-L1 expression. Imaging can be spatially colocalized with IHC (37) to determine whether origins are molecular, cellular or due to chemical/physical processes within the TME, while preclinical models can also be manipulated genetically or pharmacologically to determine how this changes the spatial and temporal expression of PD-L1 (20, 34). Overall, PD-L1 molecular imaging is expanding and holds immense potential to reveal multiple aspects of PD-L1 spatial and temporal heterogeneity, both preclinically and in patient imaging.

## Author contributions

ELB collected and reviewed the literature, prepared Figure 1, assisted with compiling Table 1, and contributed to the writing of the manuscript. RAD and AZ contributed to writing parts of this mini-review, and compiled Table 1. PMRP conceived and structured the mini-review, assisted with collecting literature, and drafted the final version of the manuscript. All authors contributed to the article and approved the submitted version.

## Funding

PMRP acknowledges the American Cancer Society (IRG-21-133-64-03), Cancer Research Foundation (P22-03203), the Elsa U. Pardee Foundation, and NIH (R01 CA244233-01A1). This research was supported by the Alvin J. Siteman Cancer Center Siteman Investment Program through the Foundation for Barnes-Jewish Hospital, Cancer Frontier Fund, and the National Cancer Institute (P30 CA091842).

## Conflict of interest

The authors declare that the research was conducted in the absence of any commercial or financial relationships that could be construed as a potential conflict of interest.

## Publisher's note

All claims expressed in this article are solely those of the authors and do not necessarily represent those of their affiliated organizations, or those of the publisher, the editors and the reviewers. Any product that may be evaluated in this article, or claim that may be made by its manufacturer, is not guaranteed or endorsed by the publisher.

## Author disclaimer

The content is solely the responsibility of the authors and does not necessarily represent the official views of the National Institutes of Health.

## References

[1] Ribas A Wolchok JD. Cancer immunotherapy using checkpoint blockade. Science. (2018) 359:1350–5. 10.1126/science.aar4060 29567705 PMC7391259

[2] Leung D Bonacorsi S Smith RA Weber W Hayes W. Molecular imaging and the PD-L1 pathway: from bench to Clinic. Front Oncol. (2021) 11:698425. 10.3389/fonc.2021.698425 34497758 PMC8420047

[3] Hegi-Johnson F Rudd S Hicks RJ De Ruysscher D Trapani JA John T. Imaging immunity in patients with cancer using positron emission tomography. npj Precis Oncol. (2022) 6:24. 10.1038/s41698-022-00263-x 35393508 PMC8989882

[4] Wu AM Pandit-Taskar N. ImmunoPET: harnessing antibodies for imaging immune cells. Mol imaging Biol. (2022) 24:181–97. 10.1007/s11307-021-01652-7 34550529

[5] McLaughlin J Han G Schalper KA Carvajal-Hausdorf D Pelekanou V Rehman J. Quantitative assessment of the heterogeneity of PD-L1 expression in non-small-cell lung cancer. JAMA Oncol. (2016) 2:46–54. 10.1001/jamaoncol.2015.3638 26562159 PMC4941982

[6] Suda K Mitsudomi T. Inter-tumor heterogeneity of PD-L1 status: is it important in clinical decision making? J Thoracic Dis. (2020) 12:1770–5. 10.21037/jtd-20-1661 32642082 PMC7330404

[7] Yi M Jiao D Xu H Liu Q Zhao W Han X. Biomarkers for predicting efficacy of PD-1/PD-L1 inhibitors. Mol Cancer. (2018) 17:129. 10.1186/s12943-018-0864-3 30139382 PMC6107958

[8] Bensch F van der Veen EL Lub-de Hooge MN Jorritsma-Smit A Boellaard R Kok IC. ^89^Zr-atezolizumab imaging as a non-invasive approach to assess clinical response to PD-L1 blockade in cancer. Nat Med. (2018) 24:1852–8. 10.1038/s41591-018-0255-8 30478423

[9] Niemeijer AN Leung D Huisman MC Bahce I Hoekstra OS van Dongen GAMS. Whole body PD-1 and PD-L1 positron emission tomography in patients with non-small-cell lung cancer. Nat Commun. (2018) 9:4664. 10.1038/s41467-018-07131-y 30405135 PMC6220188

[10] Vento J Mulgaonkar A Woolford L Nham K Christie A Bagrodia A. PD-L1 detection using ^89^Zr-atezolizumab immuno-PET in renal cell carcinoma tumorgrafts from a patient with favorable nivolumab response. J Immunother Cancer. (2019) 7:144. 10.1186/s40425-019-0607-z 31155004 PMC6545669

[11] Moroz A Lee C-Y Wang Y-H Hsiao JC Sevillano N Truillet C. A preclinical assessment of ^89^Zr-atezolizumab identifies a requirement for carrier added formulations not observed with ^89^Zr-C4. Bioconjug Chem. (2018) 29:3476–82. 10.1021/acs.bioconjchem.8b00632 30227708 PMC6430562

[12] Li M Ehlerding EB Jiang D Barnhart TE Chen W Cao T. *In vivo* characterization of PD-L1 expression in breast cancer by immuno-PET with ^89^Zr-labeled avelumab. Am J Transl Res. (2020) 12:1862–72. 32509182 PMC7270013

[13] Jagoda EM Vasalatiy O Basuli F Opina ACL Williams MR Wong K. Immuno-PET imaging of the programmed cell Death-1 ligand (PD-L1) using a zirconium-89 labeled therapeutic antibody, avelumab. Mol Imaging. (2019) 18:1536012119829986. 10.1177/1536012119829986 31044647 PMC6498777

[14] Bouleau A Lebon V Truillet C. PET imaging of immune checkpoint proteins in oncology. Pharmacol Ther. (2021) 222:107786. 10.1016/j.pharmthera.2020.107786 33307142

[15] Vivier D Sharma SK Adumeau P Rodriguez C Fung K Zeglis BM. The impact of FcγRI binding on immuno-PET. J Nucl Med. (2019) 60:1174–82. 10.2967/jnumed.118.223636 30733320 PMC6681692

[16] Smit J Borm FJ Niemeijer A-LN Huisman MC Hoekstra OS Boellaard R. PD-L1 PET/CT imaging with radiolabeled durvalumab in patients with advanced-stage non-small cell lung cancer. J Nucl Med. (2022) 63:686–93. 10.2967/jnumed.121.262473 34385342

[17] Cheng Y Shi D Xu Z Gao Z Si Z Zhao Y. ^124^I-Labeled monoclonal antibody and fragment for the noninvasive evaluation of tumor PD-L1 expression *in vivo*. Mol Pharm. (2022). 10.1021/acs.molpharmaceut.2c00084. [Epub ahead of print]. 35244407

[18] Truillet C Oh HLJ Yeo SP Lee C-Y Huynh LT Wei J. Imaging PD-L1 expression with ImmunoPET. Bioconjug Chem. (2018) 29:96–103. 10.1021/acs.bioconjchem.7b00631 29125731 PMC5773933

[19] Giesen D Broer LN Lub-de Hooge MN Popova I Howng B Nguyen M. Probody therapeutic design of ^89^Zr-CX-072 promotes accumulation in PD-L1–expressing tumors compared to normal murine lymphoid tissue. Clin Cancer Res. (2020) 26:3999–4009. 10.1158/1078-0432.CCR-19-3137 31953313

[20] Henry KE Mack KN Nagle VL Cornejo M Michel AO Fox IL. ERK inhibition improves anti-PD-L1 immune checkpoint blockade in preclinical pancreatic ductal adenocarcinoma. Mol Cancer Ther. (2021) 20:2026–34. 10.1158/1535-7163.MCT-20-1112 34349003 PMC8492510

[21] Christensen C Kristensen LK Alfsen MZ Nielsen CH Kjaer A. Quantitative PET imaging of PD-L1 expression in xenograft and syngeneic tumour models using a site-specifically labelled PD-L1 antibody. Eur J Nucl Med Mol Imaging. (2020) 47:1302–13. 10.1007/s00259-019-04646-4 31883023 PMC7101303

[22] Zhao J Wen X Li T Shi S Xiong C Wang YA. Concurrent injection of unlabeled antibodies allows positron emission tomography imaging of programmed cell death ligand 1 expression in an orthotopic pancreatic tumor model. ACS omega. (2020) 5:8474–82. 10.1021/acsomega.9b03731 32337408 PMC7178348

[23] Kelly MP Makonnen S Hickey C Arnold TC Giurleo JT Tavaré R. Preclinical PET imaging with the novel human antibody (89)Zr-DFO-REGN3504 sensitively detects PD-L1 expression in tumors and normal tissues. J Immunother cancer. (2021) 9:2025. 10.1136/jitc-2020-002025 33483343 PMC7831708

[24] Xu M Han Y Liu G Xu Y Duan D Liu H. Preclinical study of a fully human anti-PD-L1 antibody as a theranostic agent for cancer immunotherapy. Mol Pharm. (2018) 15:4426–33. 10.1021/acs.molpharmaceut.8b00371 30133286

[25] Xenaki KT Oliveira S van Bergen en Henegouwen PMP. Antibody or antibody fragments: implications for molecular imaging and targeted therapy of solid tumors. Front Immunol. (2017) 8:1287. 10.3389/fimmu.2017.01287 29075266 PMC5643388

[26] Wissler HL Ehlerding EB Lyu Z Zhao Y Zhang S Eshraghi A. Site-specific immuno-PET tracer to image PD-L1. Mol Pharm. (2019) 16:2028–36. 10.1021/acs.molpharmaceut.9b00010 30875232 PMC6521698

[27] Lv G Sun X Qiu L Sun Y Li K Liu Q. PET imaging of tumor PD-L1 expression with a highly specific nonblocking single-domain antibody. J Nucl Med. (2020) 61:117–22. 10.2967/jnumed.119.226712 31253743 PMC6954462

[28] Bridgwater C Geller A Hu X Burlison JA Zhang H-G Yan J. ^89^Zr-labeled anti-PD-L1 antibody fragment for evaluating *in vivo* PD-L1 levels in melanoma mouse model. Cancer Biother Radiopharm. (2020) 35:549–57. 10.1089/cbr.2019.3056 32315549 PMC7578182

[29] Li D Cheng S Zou S Zhu D Zhu T Wang P. Immuno-PET imaging of ^89^Zr labeled anti-PD-L1 domain antibody. Mol Pharm. (2018) 15:1674–81. 10.1021/acs.molpharmaceut.8b00062 29502426

[30] Dong H Zhu G Tamada K Chen L. B7-H1, a third member of the B7 family, co-stimulates T-cell proliferation and interleukin-10 secretion. Nat Med. (1999) 5:1365–9. 10.1038/70932 10581077

[31] Freeman GJ Long AJ Iwai Y Bourque K Chernova T Nishimura H. Engagement of the Pd-1 immunoinhibitory receptor by a novel B7 family member leads to negative regulation of lymphocyte activation. J Exp Med. (2000) 192:1027–34. 10.1084/jem.192.7.1027 11015443 PMC2193311

[32] Noguchi T Ward JP Gubin MM Arthur CD Lee SH Hundal J. Temporally distinct PD-L1 expression by tumor and host cells contributes to immune escape. Cancer Immunol Res. (2017) 5:106–17. 10.1158/2326-6066.CIR-16-0391 28073774 PMC5510474

[33] Bassanelli M Sioletic S Martini M Giacinti S Viterbo A Staddon A. Heterogeneity of PD-L1 expression and relationship with biology of NSCLC. Anticancer Res. (2018) 38:3789–96. 10.21873/anticanres.12662 29970498

[34] Kelly RJ Zaidi AH Smith MA Omstead AN Kosovec JE Matsui D. The dynamic and transient immune microenvironment in locally advanced esophageal adenocarcinoma post chemoradiation. Ann Surg. (2018) 268:992–9. 10.1097/SLA.0000000000002410 28806299

[35] Sun Y Tan J Miao Y Zhang Q. The role of PD-L1 in the immune dysfunction that mediates hypoxia-induced multiple organ injury. Cell Commun Signal. (2021) 19:76. 10.1186/s12964-021-00742-x 34256773 PMC8276205

[36] Janjigian YY Kawazoe A Yañez P Li N Lonardi S Kolesnik O. The KEYNOTE-811 trial of dual PD-1 and HER2 blockade in HER2-positive gastric cancer. Nature. (2021) 600:727–30. 10.1038/s41586-021-04161-3 34912120 PMC8959470

[37] Rüschoff JH Ferraro DA Muehlematter UJ Laudicella R Hermanns T Rodewald A-K. What's behind (68)Ga-PSMA-11 uptake in primary prostate cancer PET?. Investigation of histopathological parameters and immunohistochemical PSMA expression patterns. Eur J Nucl Med Mol Imaging. (2021) 48:4042–53. 10.1007/s00259-021-05501-1 34386839 PMC8484204

[38] Kok IC Hooiveld JS van de Donk PP Giesen D van der Veen EL Lub-de Hooge MN. ^89^Zr-pembrolizumab imaging as a non-invasive approach to assess clinical response to PD-1 blockade in cancer. Ann Oncol Off J Eur Soc Med Oncol. (2022) 33:80–8. 10.1016/j.annonc.2021.10.213 34736925

[39] Ehlerding EB Lee HJ Barnhart TE Jiang D Kang L McNeel DG. Noninvasive imaging and quantification of radiotherapy-induced PD-L1 upregulation with (89)Zr-Df-Atezolizumab. Bioconjug Chem. (2019) 30:1434–41. 10.1021/acs.bioconjchem.9b00178 30973703 PMC6521689

[40] Dhiraj K Akhilesh M Ala L Rakeeb K Sagar S Donika P. Pharmacodynamic measures within tumors expose differential activity of PD(L)-1 antibody therapeutics. Proc Natl Acad Sci USA. (2021) 118:e2107982118. 10.1073/pnas.2107982118 34508005 PMC8449349

[41] Torlakovic E Lim HJ Adam J Barnes P Bigras G Chan AWH. “Interchangeability” of PD-L1 immunohistochemistry assays: a meta-analysis of diagnostic accuracy. Mod Pathol. (2020) 33:4–17. 10.1038/s41379-019-0327-4 31383961 PMC6927905

[42] Adam J Le Stang N Rouquette I Cazes A Badoual C Pinot-Roussel H. Multicenter harmonization study for PD-L1 IHC testing in non-small-cell lung cancer. Ann Oncol. (2018) 29:953–8. 10.1093/annonc/mdy014 29351573

